# Synthesis of Reusable Silica Nanosphere-Supported Pt(IV) Complex for Formation of Disulfide Bonds in Peptides

**DOI:** 10.3390/molecules22020338

**Published:** 2017-02-22

**Authors:** Xiaonan Hou, Xiaowei Zhao, Yamei Zhang, Aiying Han, Shuying Huo, Shigang Shen

**Affiliations:** College of Chemistry and Environmental Science, Key Laboratory of Analytical Science and Technology of Hebei Province, and the MOE Key Laboratory of Medicinal Chemistry and Molecular Diagnostics, Hebei University, No. 180, Wusi Dong Road, Baoding 071002, China; 18233272151@163.com (X.H.); wjswbys@126.com (X.Z.); 15231212708@163.com (Y.Z.); scyhbu@126.com (A.H.)

**Keywords:** monodisperse silica nanospheres, supported platinum(IV) complex, peptide, intramolecular disulfide, reusability

## Abstract

Some peptide-based drugs, including oxytocin, vasopressin, ziconotide, pramlintide, nesiritide, and octreotide, contain one intramolecular disulfide bond. A novel and reusable monodispersed silica nanosphere-supported Pt(IV) complex (SiO_2_@TPEA@Pt(IV)); TPEA: *N*-[3-(trimethoxysilyl)propyl]ethylenediamine) was synthesized via a four-step procedure and was used for the formation of intramolecular disulfide bonds in peptides. Transmission electron microscopy (TEM) and chemical mapping results for the Pt(II) intermediates and for SiO_2_@TPEA@Pt(IV) show that the silica nanospheres possess a monodisperse spherical structure and contain uniformly-distributed Si, O, C, N, Cl, and Pt. The valence state of Pt on the silica nanospheres was characterized by X-ray photoelectron spectroscopy (XPS). The Pt(IV) loaded on SiO_2_@TPEA@Pt(IV) was 0.15 mmol/g, as determined by UV-VIS spectrometry. The formation of intramolecular disulfides in six dithiol-containing peptides of variable lengths by the use of SiO_2_@TPEA@Pt(IV) was investigated, and the relative oxidation yields were determined by high-performance liquid chromatography (HPLC). In addition, peptide **1** (Ac-CPFC-NH_2_) was utilized to study the reusability of SiO_2_@TPEA@Pt(IV). No significant decrease in the relative oxidation yield was observed after ten reaction cycles. Moreover, the structure of SiO_2_@TPEA@Pt(IV) after being used for ten cycles was determined to be similar to its initial one, demonstrating the cycling stability of the complex.

## 1. Introduction

A large fraction of the peptide-based drugs available on the market, including oxytocin, vasopressin, ziconotide, pramlintide, nesiritide, and octreotide, contain one or multiple intramolecular disulfide bonds. Disulfide bonds play a crucial role in both the structural and functional properties of the peptides, providing enhanced stability, selectivity, and potency [1,2,3,4]. Thus, the efficient formation of disulfide bonds is an important step in the synthesis of peptide-based drugs. The oxidation of suitable thiol-containing peptides in solution by oxidants such as air, dimethyl sulfoxide, iodine, and hydrogen peroxide is a common procedure for disulfide formation. However, the oxidation process using these oxidants often suffers from several drawbacks, including low yield, long reaction time, and the formation of side products due to over-oxidation or the oxidation of Met, Trp, and Tyr residues [5,6,7,8,9]. Therefore, great research interest exists to develop new and efficient oxidants that are suitable for disulfide formation. In this context, some efficient oxidants, such as *trans*-[PtCl_2_(CN)_4_]^2−^ and *trans*-[PtCl_2_(en)_2_]^2+^, dihydroxyselenolane oxide (DHX^ox^), and *N*-chlorosuccinimide (NCS) have been developed and utilized for the formation of disulfide bonds in peptides [10,11,12,13,14,15,16,17,18].

Alternately, the use of solid-supported oxidants has also been investigated for disulfide formation, because these oxidants possess favorable properties like reusability, mild reaction conditions, and a “pseudo-dilution” effect. So far, three types of solid-supported oxidants, including polymer-supported Ellmans’ reagent (Clear-Ox), polymer-supported oligomethionine sulfoxide (Oxyfold reagent), and ChemMatrix-supported NCS have been developed [19,20,21,22]. It has been found that at least a ten times excess of Clear-Ox must be used because of the thiol–disulfide exchange properties in peptides [19]. While high-purity disulfide bonds have been obtained with Oxyfold reagent and ChemMatrix-supported NCS, the reusability of the two oxidants has not yet been explored [20,21].

Recently, silica nanospheres have been used successfully as a solid support in the preparation of recyclable and reusable catalysts for organic synthesis, because they possess the properties of high surface area, high thermal stability, and variability in structures [23,24,25,26,27,28]. In addition, they can be simply prepared by the Stober method and functionalized by reacting with various types of coupling agents [29,30].

In this work, we have designed and synthesized a silica nanosphere-supported platinum(IV) complex as a novel solid-supported oxidant, and have used it for disulfide formation in several dithiol-containing peptides of varying lengths. The solid-supported oxidant was characterized by transmission electron microscopy (TEM), elemental analysis, X-ray photoelectron spectroscopy (XPS), and scanning electron microscopy (SEM). Further, the oxidant can be used in various buffer solutions, exhibiting an excellent durability, and it can be reused several times without change in its morphology.

## 2. Results and Discussion

### 2.1. Synthesis and Characterization of SiO_2_@TPEA@Pt(IV)

**Scheme 1:**
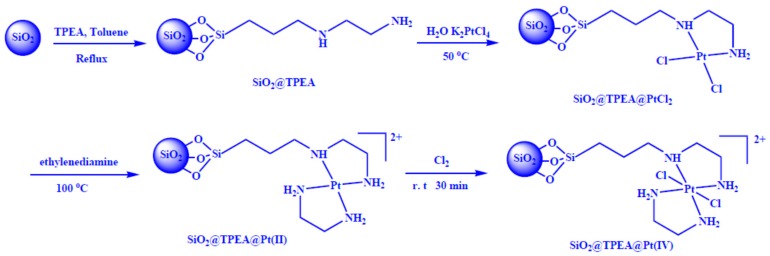
A schematic route for the synthesis of SiO_2_@TPEA@Pt(IV). TPEA: *N*-[3-(trimethoxysilyl)propyl]ethylenediamine.

SiO_2_@TPEA@Pt(IV) was synthesized via the following four-step procedure (illustrated in Scheme 1): preparation of SiO_2_@TPEA by the functionalization of SiO_2_ with *N*-[3-(trimethoxysilyl)propyl]ethylenediamine (TPEA); complexation of SiO_2_@TPEA with K_2_PtCl_4_ to give SiO_2_@TPEA@PtCl_2_; preparation of SiO_2_@TPEA@Pt(II) through the reaction of ethylenediamine with SiO_2_@TPEA@PtCl_2_; and preparation of SiO_2_@TPEA@Pt(IV) by the oxidation of SiO_2_@TPEA@Pt(II) using chlorine gas.

A TEM image of SiO_2_ is shown in Figure S1. Analysis of the image revealed that SiO_2_ possessed a monodisperse spherical structure with an average diameter of about 420 nm. After modification of SiO_2_ by TPEA, the produced SiO_2_@TPEA had a white color, and its morphology was unchanged compared to the parent SiO_2_ (see Figure S1 in Supplementary Materials). To confirm the presence of TPEA on the SiO_2_ surface, Fourier transform-infrared (FT-IR) spectra of pure SiO_2_ and SiO_2_@TPEA were compared (Figure S2); a weak band was observed at 2922.2 cm^−1^ for SiO_2_@TPEA, which was not found in the case of pure SiO_2_. This band can be assigned to the aliphatic –CH_2_ stretching vibration originated from the propyl chain in TPEA, confirming the presence of TPEA on the SiO_2_ surface [31]. Moreover, a significant reduction in the Si–OH stretching vibration peak intensity at 944.4 cm^−1^ was observed, illustrating that the surface Si–OH groups on SiO_2_ exchanged with the methoxy groups in TPEA during surface modification [32,33].

**Figure 1:**
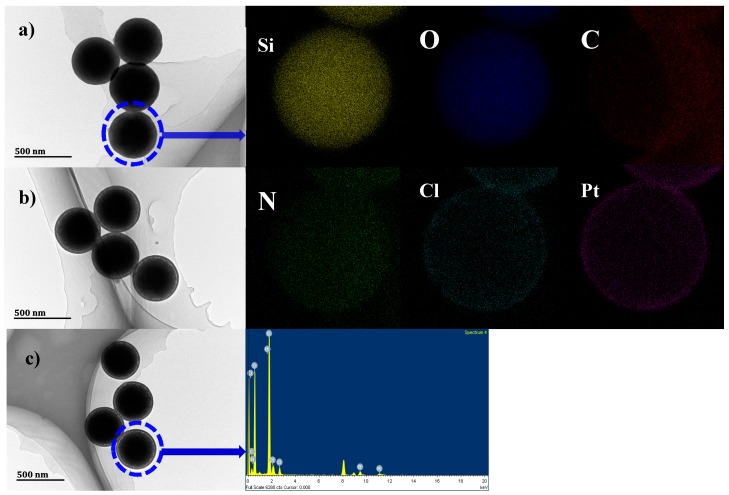
(**a**) TEM image and elemental mapping of SiO_2_@TPEA@PtCl_2_; (**b**) TEM image of SiO_2_@TPEA@Pt(II); (**c**) TEM image and energy dispersive X-ray spectroscopy (EDX) data for SiO_2_@TPEA@Pt(IV).

SiO_2_@TPEA@PtCl_2_ had brown color, and its morphology and chemical element distribution were investigated using TEM and TEM coupled with chemical mapping, respectively. As shown in Figure 1a, SiO_2_@TPEA@PtCl_2_ particles also possessed a monodisperse spherical structure. Moreover, Si, O, and C elements had a uniform distribution on the surface, confirming the presence of TPEA on the SiO_2_ nanospheres. Cl and Pt were also present on SiO_2_@TPEA@PtCl_2_, demonstrating that the –NHCH_2_CH_2_– NH_2_ group in TPEA reacted with K_2_PtCl_4_, forming SiO_2_@TPEA@PtCl_2_ [34,35]. Ethylenediamine was used to substitute the two coordinated chlorides in SiO_2_@TPEA@PtCl_2_ [36,37,38], giving rise to SiO_2_@TPEA@Pt(II). The morphology of SiO_2_@TPEA@Pt(II) is shown in Figure 1b. A corona-like structure was found for SiO_2_@TPEA@Pt(II), but this structure was not found for SiO_2_@TPEA@PtCl_2_.

For the synthesis of SiO_2_@TPEA@Pt(IV), chlorine gas was used as the oxidant because the silica nanospheres are inert to chlorine gas [39,40]. The loading of the Pt(IV) complex did not increase with increasing oxidation time. The morphology and energy dispersive X-ray spectroscopy (EDX) data of SiO_2_@TPEA@Pt(IV) are shown in Figure 1c. The EDX data suggest that the N-to-Pt atomic ratio on the surface of the material was about 4:1. The N and Pt loadings on SiO_2_@TPEA@Pt(IV) were determined by elemental analysis and inductively coupled plasma mass spectrometry (ICP-MS), respectively and were found to be 1.10 mmol and 0.21 mmol/g, respectively.

**Figure 2:**
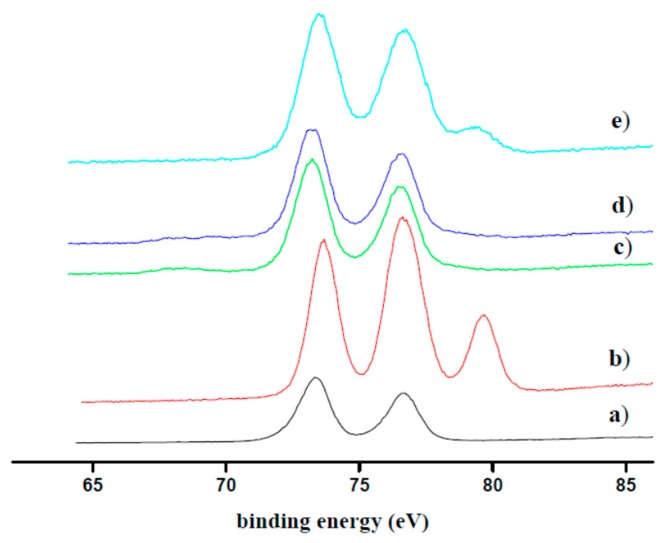
High resolution Pt 4f X-ray photoelectron spectroscopy (XPS) spectra of (**a**) Pt(en)_2_Cl_2_; (**b**) [Pt(en)_2_Cl_2_]Cl_2_; (**c**) SiO_2_@TPEA@Pt(II); (**d**) SiO_2_@TPEA@Pt(II) generated from the peptide **1** reduction of SiO_2_@TPEA@Pt(IV); and (**e**) SiO_2_@TPEA@Pt(IV).

The XPS spectra recorded for [Pt(en)_2_]Cl_2_, [PtCl_2_(en)_2_]Cl_2_, SiO_2_@TPEA@Pt(II), and SiO_2_@TPEA@Pt(IV) are shown in Figure 2. As seen in the figure, the two peaks corresponding to Pt(II)_4f(5/2)_ and Pt(II)_4f(7/2)_ are centered at binding energy values of 76.62 and 73.38 eV, respectively, in the XPS spectrum of [Pt(en)_2_]Cl_2_. In the case of [PtCl_2_(en)_2_]Cl_2_, three peaks are observed at 79.70, 76.62, and 73.63 eV. This is because [PtCl_2_(en)_2_]Cl_2_ was partially reduced by X-rays during the XPS experiments [41,42]. The peaks at 79.70 and 73.63 eV are assigned to Pt(IV)_4f(5/2)_ and Pt(II)_4f(7/2)_, respectively, whereas the peak at 76.62 eV is assigned to the overlapped Pt(II)_4f(5/2)_ and Pt(IV)_4f(7/2)_ peaks [43]. In the case of SiO_2_@TPEA@Pt(II), two peaks corresponding to Pt(II)_4f(5/2)_ and Pt(II)_4f(7/2)_ are observed at 76.56 and 73.23 eV, respectively. Compared to the XPS spectrum of [Pt(en)_2_]Cl_2_, the Pt(II)_4f(5/2)_ and Pt(II)_4f(7/2)_ peaks are shifted in the negative binding energy direction in the case of SiO_2_@TPEA@Pt(II). These negative shifts may be attributed to different complexation mechanisms in the two platinum complexes [44]. Further, three peaks at binding energies of 79.49, 76.66, and 73.483 eV are observed in the XPS spectrum of SiO_2_@TPEA@Pt(IV), which are similar to the peaks observed for [Pt(en)_2_Cl_2_]Cl_2_. Furthermore, compared to the XPS spectrum of SiO_2_@TPEA@Pt(II), a new peak at a binding energy of 79.56 eV is observed in the XPS spectrum of SiO_2_@TPEA@Pt(IV), which is assigned to Pt(IV)_4f(5/2)_. This demonstrates that SiO_2_@TPEA@Pt(II) was oxidized successfully to SiO_2_@TPEA@Pt(IV) by chlorine gas.

### 2.2. Pt(IV) Loading Determination of SiO_2_@TPEA@Pt(IV)

2,5-Dimethoxythiophenol has been used to determining the loading of immobilized NCS as described in the literature [22]. However, the reaction between [Pt(en)_2_Cl_2_]Cl_2_ and 2,5-dimethoxythiophenol has not yet been studied. Therefore, the stoichiometric ratio between *trans*-[PtCl_2_(en)_2_]^2+^ and 2,5-dimethoxythiophenol was determined in this work. A plot of absorbance versus [PtCl_2_(en)_2_^2+^] is presented in Figure S3, which shows that the data points follow two straight lines. The stoichiometric ratio of [PtCl_2_(en)_2_^2+^]/[2,5-dimethoxythiophenol] was estimated to be 1:1.8. This stoichiometric ratio implies that 2,5-dimethoxythiophenol was mainly oxidized to form a 2,5-dimethoxythiophenol dimer linked by a disulfide, which was confirmed by electrospray ionization mass spectrometry (ESI-MS) (observed [M + Na]^+^ *m*/*z* 361.2). The same oxidation product was observed when 2,5-dimethoxythiophenol was oxidized by SiO_2_@TPEA@Pt(IV). Thus, we used the reaction between 2,5-dimethoxythiophenol and an excess of [Pt(en)_2_Cl_2_]Cl_2_ to construct a calibration curve which can be employed to determine the loading of SiO_2_@TPEA@Pt(IV). The calibration curve is shown in Figure S4. The Pt(IV) complex loading was determined to be 0.15 mmol/g.

### 2.3. Intramolecular Disulfide Formation in Peptides by SiO_2_@TPEA@Pt(IV)

**Scheme 2:**
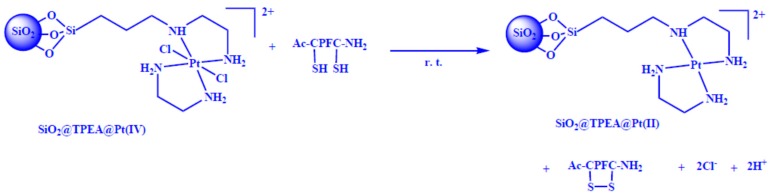
Proposed mechanism for disulfide formation in peptide **1** by SiO_2_@TPEA@Pt(IV).

**Figure 3:**
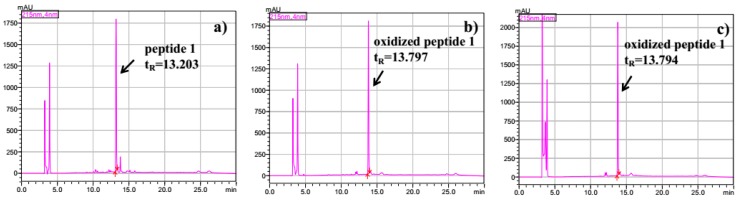
HPLC chromatograms of (**a**) peptide **1** (1.0 mg/mL, 1.5 mL) after stirring for 30 min; (**b**) the supernatant from the reaction of SiO_2_@TPEA@Pt(IV) (50 mg) with peptide **1** (1.0 mg/mL, 1.5 mL) for 30 min; and (**c**) the mixture from the reaction of [Pt(en)_2_Cl_2_]Cl_2_ (1.8 mg) with peptide **1** (1.0 mg/mL, 1.5 mL).

Dithiol-containing peptides with variable lengths that were used in this work are listed in Table 1; their corresponding oxidized forms containing a disulfide ring vary in size from 14 to 38 atoms. The result of the reaction between peptide **1** and SiO_2_@TPEA@Pt(IV) is shown in Figure 3. A comparison of the chromatograms (Figure 3a,b) shows that peptide **1** was oxidized thoroughly by SiO_2_@TPEA@Pt(IV) to its oxidized form. Details of the reaction conditions and MS spectra are provided in the Supporting Information. The reaction mechanism is illustrated in Scheme 2, similar to that proposed earlier for the reactions between [Pt(en)_2_Cl_2_]Cl_2_ and the dithiol-containing peptides [10,11,12,13,14].

Previously, *trans*-[PtCl_2_(en)_2_]^2+^ was found to be a highly selective and efficient reagent for the rapid and quantitative formation of disulfide bonds in peptides [11]; no side reactions occurred on the side chains of tryptophan, tyrosine, and methionine, and no dimers formed, though an intermolecular disulfide link was observed with the Pt(IV) complex [11]. Therefore, the peak area of isoxidized peptide generated by *trans*-[PtCl_2_(en)_2_]^2+^ oxidation was used as a reference to investigate the efficiency of SiO_2_@TPEA@Pt(IV) for the formation of disulfide bonds in peptides. The relative oxidation yield is defined as S_A_/S_B_, where S_A_ refers to the peak area corresponding to oxidized peptide **1** generated by the oxidation of SiO_2_@TPEA@Pt(IV) (Figure 3b), and S_B_ pertains to the peak area of oxidized peptide 1 generated by *trans*-[PtCl_2_(en)_2_]^2+^ (Figure 3c). The relative oxidation yield by SiO_2_@TPEA@Pt(IV) was calculated to be 84%.

**Figure 4:**
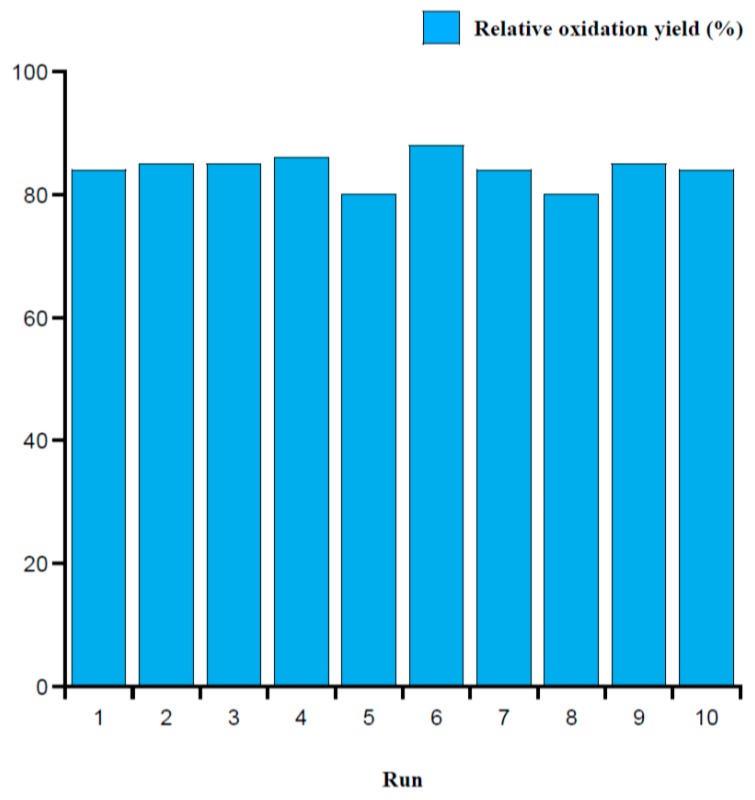
Reusability of SiO_2_@TPEA@Pt(IV) over ten reaction cycles.

**Figure 5:**
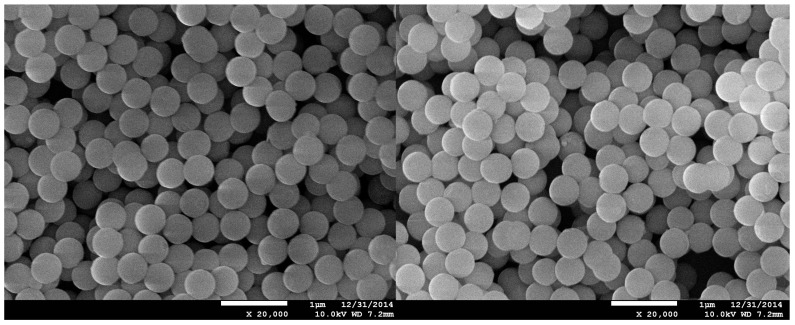
SEM images of SiO_2_@TPEA@Pt(IV) before (**left**) and after (**right**) being used for ten cycles.

The solid products of the reaction between SiO_2_@TPEA@Pt(IV) and peptide **1** were separated by centrifugation and washed with *N,N*-dimethylformamide (DMF) and water, and were then oxidized by Cl_2_, regenerating SiO_2_@TPEA@Pt(IV). The reusability of this material for the formation of intramolecular disulfide bond in peptide **1** was examined. No significant decrease in the relative oxidation yield was observed after ten run cycles (Figure 4). Moreover, the morphology of SiO_2_@TPEA@Pt(IV) before and after ten cycles was analyzed by SEM (Figure 5), showing that the SiO_2_@TPEA@Pt(IV) nanospheres were still stable after ten cycles of use. On the other hand, SiO_2_@TPEA@Pt(II) generated from the reduction of SiO_2_@TPEA@Pt(IV) by an excess of peptide **1** was characterized by XPS (Figure 2). A comparison of the XPS spectra of SiO_2_@TPEA@Pt(IV) and SiO_2_@TPEA@Pt(II) reveals that the peak at a binding energy of 79.56 eV—which is assigned to Pt(IV)_4f(5/2)_—disappeared in the latter case. This suggests that SiO_2_@TPEA@Pt(IV) was reduced thoroughly by peptide **1**.

The role of SiO_2_@TPEA@Pt(IV) in the oxidative formation of disulfide bonds in the other five peptides listed in Table 1 was also investigated. The relative oxidation yields for these peptides are given in Table 1. Figures S5–S22 in the Supplementary Materials summarize the reaction conditions, high-performance liquid chromatography (HPLC) chromatograms, and MS spectra for these reactions. We found that tryptophan, tyrosine, and methionine residues were not modified by this oxidant under the conditions used in present work. Additionally, no dimers were observed in the reactions. Thus, a good selectivity and conversion were obtained for all the peptides. On the other hand, SiO_2_@TPEA@Pt(IV) can be very readily separated from the peptide, and its regeneration is easier than that of *trans*-[PtCl_2_(en)_2_]^2+^ [36,45].

With reduced oxytocin (peptide **5** in Table 1) as an example, the oxidation property of oxytocin by SiO_2_@TPEA@Pt(IV) was further investigated. The results are shown in Figure S17a–d. The HPLC chromatograms in Figure S17a,b show that the reduced oxytocin was not significantly oxidized by air or by SiO_2_@TPEA@Pt(II). Moreover, the peak areas corresponding to reduced oxytocin were essentially the same, demonstrating that the reduced oxytocin is not adsorbed onto SiO_2_@TPEA@Pt(II). The HPLC chromatograms for the reactions between the reduced oxytocin and SiO_2_@TPEA@Pt(IV), as well as *trans*-[PtCl_2_(en)_2_]^2+^, are presented in Figure S17c,d, showing that there were no significant byproducts formed when the reduced oxytocin was oxidized to oxytocin by SiO_2_@TPEA@Pt(IV). On the other hand, the peak area of oxytocin in Figure S23a was similar to that of oxytocin in Figure S23b, demonstrating that SiO_2_@TPEA@Pt(II) did not adsorb oxytocin under the reaction conditions.

The oxidation yield was also investigated using the reaction between reduced oxytocin (40 mg) and SiO_2_@TPEA@Pt(IV) (0.65 g). After the completion of the reaction, pure oxytocin (24 mg) was obtained as the trifluoroacetic acid (TFA) salt. Thus, the overall disulfide bond formation yield was 60%. On the other hand, the nanospheres containing SiO_2_@TPEA@Pt(IV) and SiO_2_@TPEA@Pt(II) were washed with pH 4.5 buffer and water. Some sulfur-containing compounds were found to be adsorbed onto the nanospheres, and could only be removed using DMF. Unfortunately, the structures of the sulfur-containing compounds are unknown. These sulfur-containing compounds may cause the reaction between SiO_2_@TPEA@Pt(IV) and reduced oxytocin to exhibit a lower yield than the reaction between *trans*-[PtCl_2_(en)_2_]^2+^ and reduced oxytocin.

The reaction between SiO_2_@TPEA@Pt(IV) and peptide **5** in different pH buffer solutions (pH 2.0 and 7.1) was also investigated. The relative oxidation yields were between 57% and 65%, which indicate that SiO_2_@TPEA@Pt(IV) can also be used for the formation of disulfides in peptides in different pH buffers.

The yields for the formation of intramolecular disulfide bonds through the oxidation of dithiol-containing peptides by different solid-supported oxidants have been investigated in the literature [15,16]. For example, the oxidation yield in the case of somatostatin was 30% when supported Ellman’s reagent was used, because a significant amount of peptides was covalently bound to resin beads owing to the disulfide exchange mechanism. For Oxyfold reagent, the overall oxidation yield for the synthesis of disulfide bonds in a model peptide (sequence: H-LCAGPCL-NH_2_) was 57%. The oxidation yield was not reported when ChemMatrix-supported NCS was used as the oxidant [22]. In the present work, the oxidation yield for disulfide formation in oxytocin was 60% (the relative oxidation yield was 59%).

## 3. Experimental Procedures

### 3.1. Materials

Monodispersed silica nanospheres (SiO_2_) were obtained as a gift from Dr. Cuimiao Zhang (Hebei University). Fmoc-protected amino acids, Fmoc-Rink-amide-AM resin, and *O*-(benzotriazol-1-yl)-*N*,*N*,*N’*,*N’*-tetramethyluronium tetrafluoroborate (HBTU) were purchased from GL Biochem (Shanghai, China). Diisopropylethylamine (DIEA), piperidine, triisopropylsilane, *N*-[3-(trimethoxysilyl)propyl]ethylenediamine (TPEA), 2,5-dimethoxythiophenol, and [Pt(en)_2_]Cl_2_ were purchased from Sigma-Aldrich. Trifluoroacetic acid (TFA), *N*,*N*-dimethylformamide (DMF), phenol, K_2_PtCl_4_, ethylenediamine, and acetonitrile were purchased from Adamas (Tansoole, Shanghai, China). Acetic acid, sodium acetate, ethanol, concentrated HCl, and KMnO_4_ were purchased from Shanghai Chemical Reagent Company (Shanghai, China). [PtCl_2_(en)_2_]Cl_2_ was synthesized according to a method published in the literature [35]. The UV-VIS spectrum of the [PtCl_2_(en)_2_]Cl_2_ solution prepared in this study is in excellent agreement with that reported earlier for *trans*-[PtCl_2_(en)_2_]^2+^ [35].

### 3.2. Instrumentation

TEM images of SiO_2_ and SiO_2_@TPEA, and EDX data and elemental mapping of SiO_2_@TPEA@PtCl_2_, SiO_2_@TPEA@Pt(II), and SiO_2_@TPEA@Pt(IV) were recorded on a JEM 2100F transmission electron microscope (JEOL Ltd., Tokyo, Japan) at Beihang University. Elemental analysis was conducted to determine the N loading using a CE-440 elemental analyzer (Exeter Analytical Inc., North Chelmsford, MA, USA). The Pt loading on SiO_2_@TPEA@Pt(IV) was determined by an X Series 2 ICP-MS system (Thermo Fisher Scientific Inc., Waltham, MA, USA). The loading of Pt(IV) complex was determined using a TU-1950 spectrophotometer (Beijing Puxi Inc., Beijing, China) with 1.0 cm quartz cells. SEM images were recorded on a JSM-7500F cold field scanning electron microscope (JEOL, Ltd., Tokyo, Japan). Fourier transform-infrared (FT-IR) spectra were recorded on a VERTEX 70 FT-IR spectrometer (Bruker Inc., Ettlingen, Germany) with the KBr pellet technique. X-ray photoelectron spectroscopy (XPS) data for SiO_2_@TPEA@Pt(IV) nanospheres were collected on an ESCALAB 250 Xi X-ray photoelectron spectrometer (Thermo Fisher Scientific Inc.). The peptides were synthesized by the use of a Focus XC solid phase peptide synthesizer (AAPPTec, Louisville, KY, USA) and purified on an LC-6AD semi-preparative high-performance liquid chromatography (HPLC) system (Shimadzu, Kyoto, Japan) and analyzed on an LC-20AB HPLC system (Shimadzu). Mass spectra for product analysis were recorded on an Agilent 1200/6310 electrospray ionization mass spectrometry (Agilent Technologies, Santa Clara, CA, USA) and on a Bruker Apex Ultra electrospray mass spectrometer (Bruker Daltonics Inc., Billerica, MA, USA).

### 3.3. Preparation of TPEA-Modified Silica Nanospheres (SiO_2_@TPEA)

SiO_2_@TPEA was prepared according to a procedure available in the literature [31]. Typically, 1.5 g of SiO_2_ was added to 50 mL of dry toluene, and the mixture was ultrasonicated for 1 h to obtain a suspension. To the suspension, 2 mL of TPEA was added, and the mixture was refluxed for 10 h with stirring under N_2_ atmosphere. After cooling to room temperature, the resulting amine-functionalized silica nanospheres (SiO_2_@TPEA) were separated by centrifugation, washed with dry toluene and ethanol, and dried under vacuum.

### 3.4. Synthesis of SiO_2_@TPEA@Pt(IV)

Firstly, 1.4 g of SiO_2_@TPEA was suspended in 50 mL water and ultrasonicated for 1 h. The mixture was heated at 50 °C with stirring under N_2_ atmosphere. An aqueous solution of K_2_PtCl_4_ (0.15 M) was added drop-wise until the supernatant had a slightly yellow color. The resulting platinum complex-modified silica nanospheres (SiO_2_@TPEA@PtCl_2_) were centrifuged and washed with water several times to remove the unreacted K_2_PtCl_4_. Next, the SiO_2_@TPEA@PtCl_2_ obtained above was re-dispersed in 50 mL water and ultrasonicated for 30 min; 3 mL of ethylenediamine was added, and the resulting mixture was stirred at 100 °C for 5 h under N_2_ atmosphere. The solid product—denoted as SiO_2_@TPEA@Pt(II)—was centrifuged and washed with water and ethanol, and then dried under vacuum. Finally, 50 mg of SiO_2_@TPEA@Pt(II) was suspended in 10 mL of HCl solution (10 mM), and the mixture was ultrasonicated for 10 min. Chlorine gas (generated by the reaction of KMnO_4_ with concentrated HCl) was bubbled through the mixture at room temperature for 30 min with stirring, following which N_2_ gas was bubbled for an additional 1 h. The silica nanosphere-supported Pt(IV) complex (SiO_2_@TPEA@Pt(IV)) was centrifuged, washed thoroughly with water and ethanol, and dried under vacuum.

### 3.5. Determination of Pt(IV) Loading in the Synthesized SiO_2_@TPEA@Pt(IV)

#### 3.5.1. Stoichiometry and Product Analysis

The stoichiometry of the reaction between *trans*-[PtCl_2_(en)_2_]^2+^ and 2,5-dimethoxythiophenol was determined in a solution containing a mixture of pH 4.5 buffer and acetonitrile (1:3 *v*/*v*) using a TU-1950 spectrophotometer. In these experiments, a series of solutions containing 0.1 mM 2,5-dimethoxythiophenol and various concentrations of *trans*-[PtCl_2_(en)_2_]^2+^ (1.96 × 10^−2^ mM to 0.118 mM) were prepared and maintained for 1 h at 25 °C. The absorbance values of these solutions were then determined at a wavelength of 330 nm, and the stoichiometry was derived from plots of absorbance versus [PtCl_2_(en)_2_^2+^]. To analyze the product obtained from the reaction between 2,5-dimethoxythiophenol and *trans*-[PtCl_2_(en)_2_]^2+^, the reaction mixture composed of 1.0 mM *trans*-[PtCl_2_(en)_2_]^2+^ and 2.0 mM 2,5-dimethoxythiophenol was dissolved in a solution containing a mixture of pH 4.5 buffer and acetonitrile (1:3 *v*/*v*) and maintained for 1 h at 25 °C. This solution was then analyzed using an ESI-MS spectrometer.

#### 3.5.2. Determination of Pt(IV) Loading in SiO_2_@TPEA@Pt(IV)

All of the solutions were prepared in a pH 4.5 buffer/acetonitrile (1:3 *v*/*v*) mixture. 2,5-dimethoxythiophenol solution (0.5 mL, 20.0 mM) was mixed with *trans*-[PtCl_2_(en)_2_]^2+^ (2.5 mL, 3 mM), and diluted to 5.0 mL. After reaction for 1 h, the mixture was further diluted to 2,5-dimethoxythiophenol disulfide concentrations of 0.025, 0.05, 0.10, 0.15, 0.20, 0.25, and 0.3 mM, and absorbance values were measured at 330 nm. The experiments were performed in triplicate, and a calibration curve of absorbance as a function of concentration was prepared. Next, SiO_2_@TPEA@Pt(IV) (25 mg) was placed in a solution containing 2,5-dimethoxythiophenol (2 mL, 5.0 mM), and the mixture was shaken for 1 h at room temperature and centrifuged. The supernatant (0.5 mL) was diluted to 5 mL. An absorbance value of 0.56 was obtained, corresponding to a Pt(IV) loading of 0.15 mmol/g.

### 3.6. Disulfide Bond Formation in Peptides

#### 3.6.1. Synthesis and Purification of Peptides

Peptides were synthesized by means of a Focus XC solid phase peptide synthesizer using the standard Fmoc methodology [46]. Fmoc-Rink-amide resin (0.66 mmol/g, 250 mg) was used for the synthesis of the peptides. All of the coupling reactions were carried out using 3 mL of amino acid (0.33 mM) in DMF, 3 mL of HBTU (0.33 M) in DMF, and 2 mL of DIEA (1.0 M) in DMF for 50 min. Fmoc deprotection was performed with a 20% piperidine DMF solution. A cleavage cocktail containing 4% phenol, 2% water, 2% triisopropylsilane, and 92% TFA was used to cleave the peptides from the resin. After the resin was removed by filtration, the filtrate was treated with diethyl ether, and the peptides were separated by centrifugation and dissolved in water. Crude peptides were obtained after lyophilization. The peptides were purified by a semi-preparative reverse phase (RP)-HPLC system equipped with a UV-VIS detector at 215 nm using a 250 mm × 20 mm ODS-C_18_ column at a flow rate of 10 mL/min. Two solvent systems consisting of 0.03% TFA in acetonitrile and 0.03% TFA in water (referred to as solvents A and B) were used for peptide elution with a suitable gradient. After lyophilization, the peptides were obtained as TFA salts and used for further experiments.

#### 3.6.2. General Method of Disulfide Formation

An excess of SiO_2_@TPEA@Pt(IV) was added to a peptide solution, and the mixture was stirred for 30 min at 25 °C, followed by centrifugation to remove the nanospheres. The obtained solution was analyzed using a gradient RP-HPLC system equipped with a UV-VIS detector at 215 nm using a 250 mm × 4.6 mm C_8_ column at a flow rate of 1.0 mL/min. The solvent system encompassed A (0.03% or 0.1% TFA in acetonitrile) and B (0.03% or 0.1% TFA in water).

## 4. Conclusions

SiO_2_@TPEA@Pt(IV) was successfully synthesized and used for the formation of disulfide in six dithiol-containing peptides. The concept of “relative oxidation yield” was defined and used to evaluate the efficiency of disulfide formation in these peptides. SiO_2_@TPEA@Pt(IV) was stable and could be used for at least ten cycles without any decrease in the relative oxidation yield. The overall yield was 60% for the oxidative synthesis of oxytocin by this oxidant. However, the reason for the low yield during the synthesis of oxytocin is unclear. We intend to investigate the reason for the low yield in the future, which can aid in the efficient synthesis of disulfide bonds in peptides.

## Figures and Tables

**Table 1:** The sequences of dithiol-containing peptides and the relative oxidation yields.

Peptide Sequence	Relative Oxidation Yield
1 Ac-CPFC-NH_2_	84%
2 CGYCHKLHQMK-NH_2_	68%
3 CYFQNCPRG-NH_2_ (reduced arginine vasopressin)	68%
4 CRGDKGPDC-NH_2_ (reduced iRGD peptide)	50%
5 CYINQCPLG-NH_2_ (reduced oxytocin)	59%
6 AGCKNFFWKTFTSC (reduced somatostatin)	50%

## Supplementary Materials

The supplementary materials are available online.
