# Supplementary material for: Synthesis of Reusable Silica Nanosphere-Supported Pt(IV) Complex for Formation of Disulfide Bonds in Peptides
Source: Molecules. 2017 Feb 22;22(2):338. doi: 10.3390/molecules22020338 (PMC6155793; doi:10.3390/molecules22020338)
Supplement: Supplementary file 1 [file molecules-22-00338-s001.pdf]

Supplementary Materials

Synthesis of Reusable Silica Nanosphere-Supported Pt(IV) Complex for Formation of Disulfide Bonds in Peptides

Xiaonan Hou, Xiaowei Zhao, Yamei Zhang, Aiying Han, Shuying Huo*, and Shigang Shen*

College of Chemistry and Environmental Science, Key Laboratory of Analytical Science and Technology of Hebei Province, and MOE Key Laboratory of Medicinal Chemistry and Molecular Diagnostics, Hebei University, Baoding 071002, Hebei Province, P. R. China

1. TEM images of SiO_2 and SiO_2@TPEA.

2. Infrared spectra of pure $SiO_2$and SiO_2@TPEA.

3. Determination of Pt(IV) complex loading on SiO_2@TPEA@Pt(IV)

4. Disulfide bond formation in peptides

1. TEM images of SiO_2 and SiO_2@TPEA

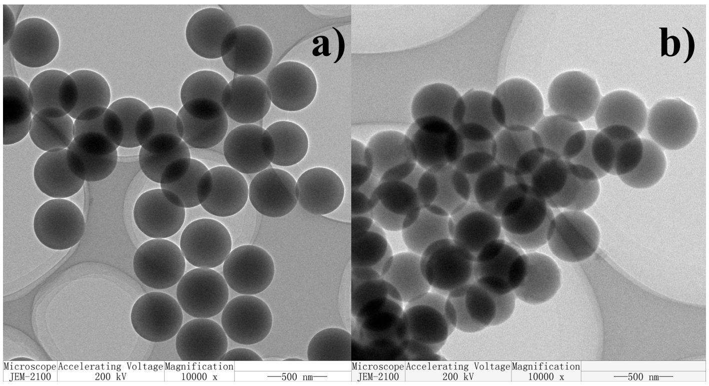

Figure S1. TEM images of (**a**) SiO_2 and (**b**) SiO_2@TPEA. TPEA: *N*-[3-(trimethoxysilyl)propyl]ethylenediamine.

2. Infrared spectra of pure SiO_2 and SiO_2@TPEA

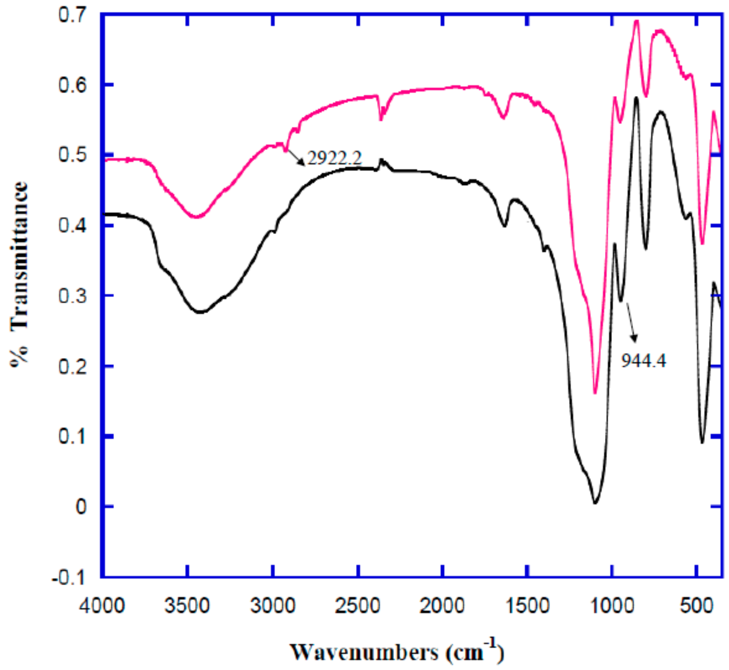

Figure S2. Infrared spectra of pure SiO_2 (—) and SiO_2@TPEA (—).

3. Determination of Pt(IV) complex loading on SiO_2@TPEA@Pt(IV)

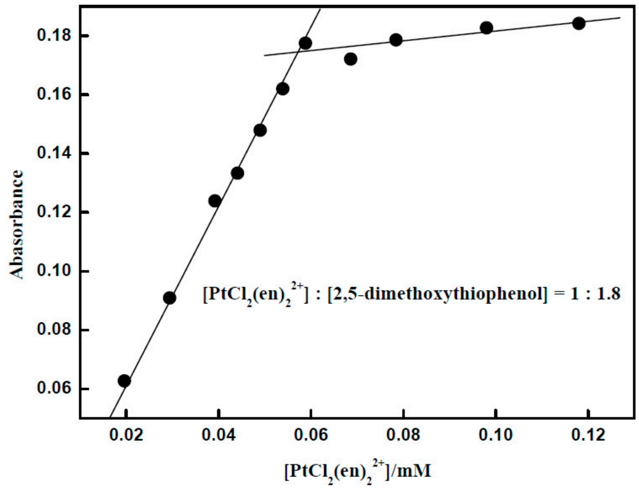

Figure S3. Absorbance at 330 nm for a series of reaction mixtures, in which the concentration of *trans*-$[PtCl_2(en)_2]^{2+}$ varied from 1.96 × 10^{-2} mM to 0.118 mM and 2,5-dimethoxythiophenol was kept constant at 0.10 mM. The reaction medium was a pH 4.5 buffer solution at 25 °C.

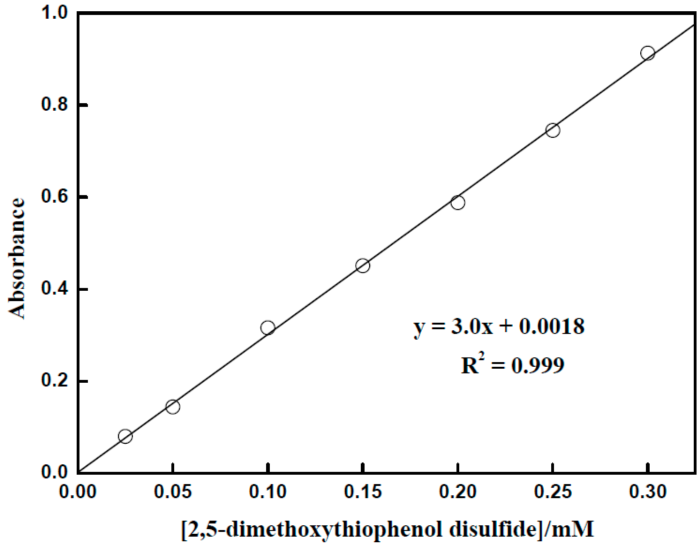

Figure S4. Calibration curve for the determination of Pt(IV) complex loading in the synthesized SiO_2@TPEA@Pt(IV).

4. Disulfide bond formation in peptides

4.1. HPLC and electrospray ionization mass spectrometry (ESI-MS) characterization of peptide 1

Peptide 1 was dissolved in a pH 4.5 buffer (1 mg/mL, 1.5 mL) and stirred at 25 °C for 30 min. The mixture was then analyzed by an HPLC system equipped with a UV-vis detector at 215 nm using a 250 mm × 4.6 mm C_8 column at a flow rate of 1.0 mL/min at room temperature. The HPLC solvent consisted of a mixture of solvent A (0.03% trifluoroacetic acid (TFA) in acetonitrile) and solvent B (0.03% TFA in water). The elution protocol for analytical HPLC was as follows: 90% B, followed by a linear gradient to 60% B over 10 min and further to 50% B over 5 min, held at 50% B for 5 min, and finally returned to 90% over 5 min. The HPLC chromatogram obtained is shown in Figure S5a.

Next, to the mixture of peptide **1** in a pH 4.5 buffer (1 mg/mL, 1.5 mL), SiO_2@TPEA@Pt(IV) (50 mg) or $[Pt(en)_2Cl_2]Cl_2$ (1.8 mg) were added with stirring at 25 °C for 30 min. The supernatant obtained after centrifugation in the former case and the reaction mixture in the latter case were analyzed using the HPLC method described above. The HPLC chromatograms obtained for the two cases are presented in Figure S5b and S5c, respectively.

Peptide **1** as well as its oxidation product were also characterized by ESI-MS in the positive mode observed $[M + H^+]^+$ *m/z* 510.18 and $[M + Na^+]^+$ *m/z* 532.17 for peptide **1** (Figure S6) and $[M + H^+]^+$ *m/z* 408.17 and $[M + Na^+]^+$ *m/z* 530.15 for

oxidized peptide **1** (Figure S7).

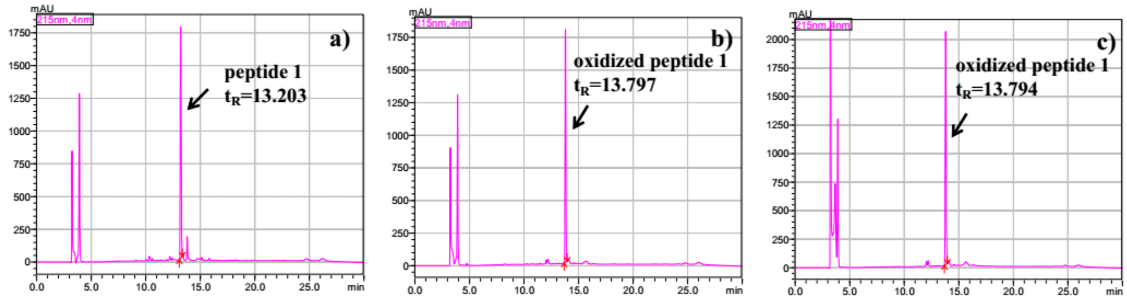

Figure S5. HPLC chromatograms of (**a**) peptide **1** (1.0 mg/mL, 1.5 mL) after stirring for 30 min; (**b**) supernatant from the reaction of SiO_2@TPEA@Pt(IV) (50 mg) with peptide **1** (1.0 mg/mL, 1.5 mL) for 30 min; and (**c**) mixture from the reaction of $[Pt(en)_2Cl_2]Cl_2$ (1.8 mg) with peptide **1** (1.0 mg/mL, 1.5 mL).

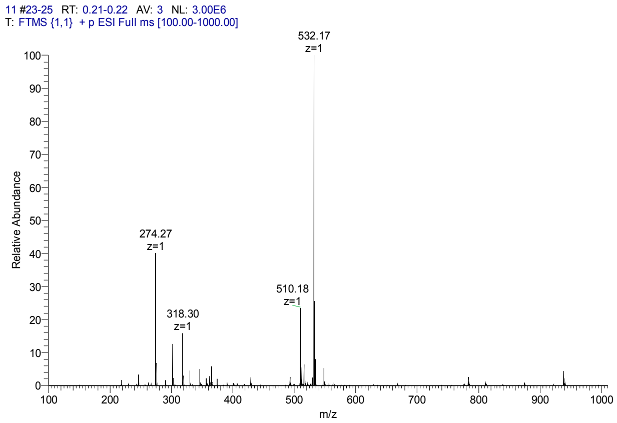

Figure S6. MS of reduced peptide **1**.

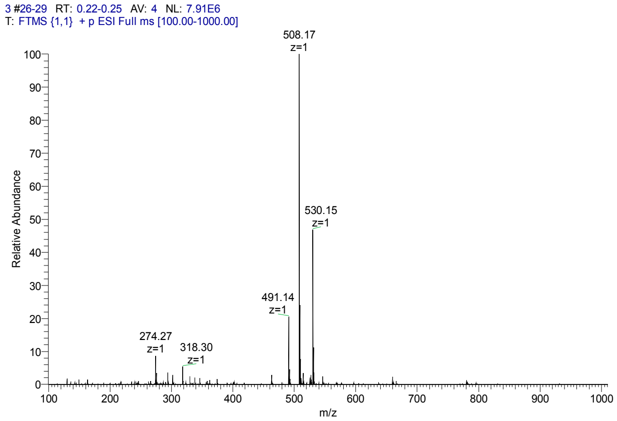

Figure S7. MS of oxidized peptide **1**.

4.2. HPLC and ESI-MS characterization of peptide 2

Peptide **2** was dissolved in a pH 4.5 buffer (1 mg/mL, 1.5 mL) and stirred at 25 °C for 80 min. The mixture was then analyzed by an HPLC system equipped with a UV-vis detector at 215 nm using a 250 mm × 4.6 mm C_8 column at a flow rate of 1.0 mL/min at room temperature. The HPLC solvent consisted of solvent A (0.1% TFA in acetonitrile) and solvent B (0.1% TFA in water). The elution protocol for analytical HPLC started with 90% B, followed by a linear gradient to 70% B over 22 min. The HPLC chromatogram obtained is shown in Figure S8a.

Next, to the mixture of peptide **2** in a pH 4.5 buffer (1 mg/mL, 1.5 mL), SiO_2@TPEA@Pt(IV) (7.5 mg) or $[Pt(en)_2Cl_2]Cl_2$ (0.9 mg) were added with stirring at 25 °C for 80 min. The supernatant obtained after centrifugation in the former case and the reaction mixture in the latter case were analyzed using the HPLC method described above. The HPLC chromatograms obtained for the two cases are presented in Figure S8b and S8c, respectively.

Peptide **2** as well as its oxidation product were also characterized by ESI-MS in the positive mode observed $[M + H^+]^+$ *m/z* 1346.63 and $[M + 2H^+]^{2+}$ *m/z* 673.82 for peptide **2** (Figure S9) and $[M + H^+]^+$ *m/z* 1344.61 and $[M + 2H^+]^{2+}$ *m/z* 672.81 for oxidized peptide **2** (Figure S10).

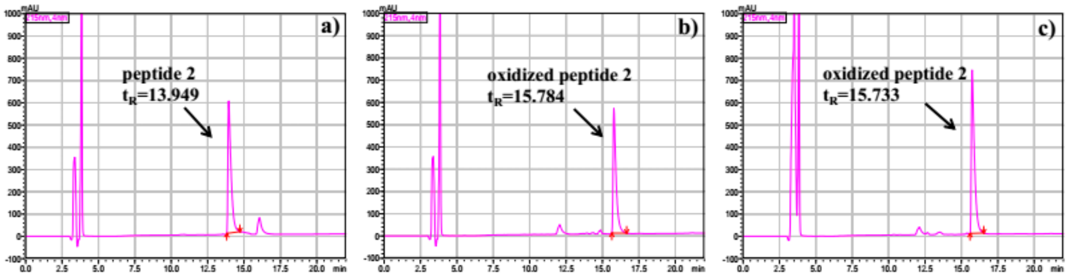

Figure S8. HPLC chromatograms of (**a**) peptide **2** after stirring for 80 min; (**b**) supernatant from the reaction between SiO_2@TPEA@Pt(IV) and peptide **2** after reaction for 80 min; and (**c**) $[Pt(en)_2Cl_2]Cl_2$ and peptide **2** reaction mixture.

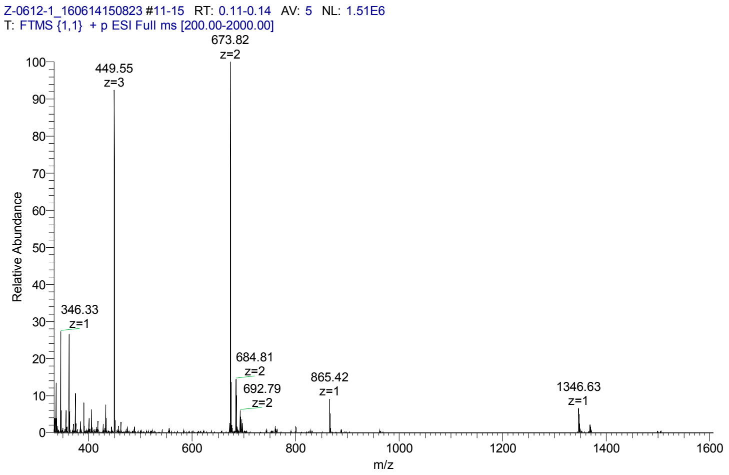

Figure S9. MS of reduced peptide **2**.

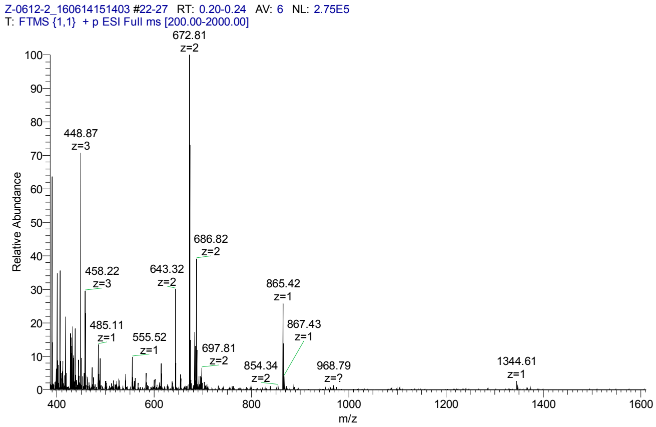

Figure S10. MS of oxidized peptide **2**.

4.3. HPLC and ESI-MS characterization of reduced arginine vasopressin

Reduced arginine vasopressin was dissolved in a pH 4.5 buffer (2 mg/mL, 1.5 mL) and stirred at 25 °C for 30 min. The mixture was then analyzed using HPLC equipped with a UV-vis detector at 215 nm with a 250 mm × 4.6 mm C_8 column at a flow rate of 1.0 mL/min at room temperature. The HPLC elution solvent consisted of 20% solvent A (0.03% TFA in acetonitrile) and 80% solvent B (0.03% TFA in water). The HPLC chromatogram obtained is shown in Figure S11a.

Next, reduced arginine vasopressin was dissolved in a pH 4.5 buffer (2 mg/mL, 1.5 mL) and reacted with SiO_2@TPEA@Pt(IV) (50 mg) or $[Pt(en)_2Cl_2]Cl_2$ (1.7 mg) under stirring at 25 °C for 30 min. In the former case, the mixture was subjected to centrifugation and the supernatant obtained was analyzed using HPLC (Figure S11b). In the latter case, the reaction mixture was analyzed by HPLC (Figure S11c) using the conditions described above.

Reduced arginine vasopressin and arginine vasopressin were characterized by ESI-MS in the positive mode. In the case of arginine vasopressin, the observed $[M + H^+]^+$ *m/z* was 1086.46 and $[M + 2H^+]^{2+}$ *m/z* was 543.73 (Figure S12). In the case of arginine vasopressin, the observed $[M + H^+]^+$ *m/z* was 1084.45 and $[M + 2H^+]^{2+}$ *m/z* was 542.73 (Figure S13).

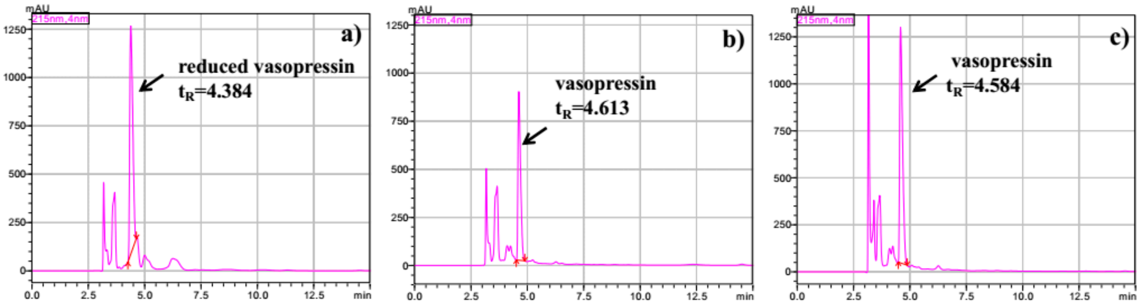

Figure S11. HPLC chromatograms of (**a**) reduced arginine vasopressin after stirring for 30 min; (**b**) supernatant from the reaction of SiO_2@TPEA@Pt(IV) with reduced arginine vasopressin for 30 min; and (**c**) $[Pt(en)_2Cl_2]Cl_2$ and reduced arginine vasopressin reaction mixture.

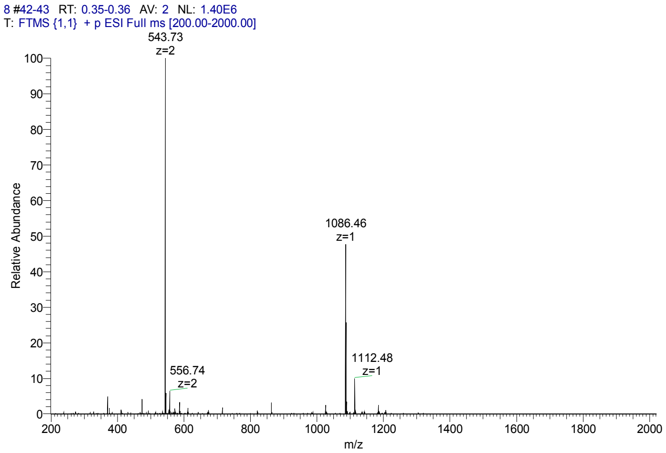

Figure S12. MS of reduced arginine vasopressin.

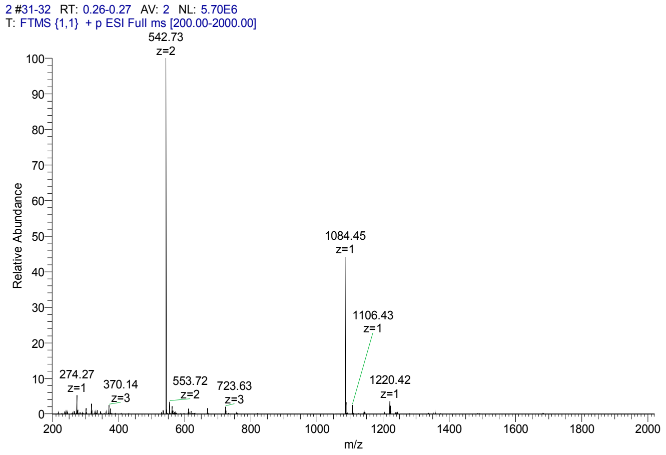

Figure S13. MS of arginine vasopressin.

4.4. HPLC and ESI-MS characterization of reduced iRGD (a disulfide-based cyclic RGD peptide, c(CRGDKGPDC)) peptide

Reduced iRGD peptide was dissolved in a pH 4.5 buffer (2 mg/mL, 1.2 mL) and stirred at 25 °C for 30 min. The solution was analyzed by a gradient HPLC equipped with a UV-vis detector at 215 nm using a 250 mm × 4.6 mm C_8 column at a flow rate of 1.0 mL/min at room temperature. The HPLC solvent consisted of solvent A (0.1% TFA in acetonitrile) and solvent B (0.1% TFA in water). The elution protocol for analytical HPLC started with 95% B, followed by a linear gradient to 92% B over 20 min, and back to 95% over 5 min. The HPLC chromatogram obtained is shown in Figure S14a.

Next, the reduced iRGD peptide was dissolved in a pH 4.5 buffer (2 mg/mL, 1.2 mL) and reacted with SiO_2@TPEA@Pt(IV) (40 mg) or $[Pt(en)_2Cl_2]Cl_2$ (1.5 mg) under stirring at 25 °C for 30 min. In the former case, the supernatant was obtained after centrifugation and analyzed using HPLC, whereas in the latter case, the reaction mixture was analyzed using HPLC with the conditions described above. The results are shown in Figure S14b and Figure S14c, respectively.

Reduced iRGD and iRGD were characterized by ESI-MS in the positive mode; for reduced iRGD peptide, observed $[M + H^+]^+$ *m/z* 949.40 and $[M + 2H^+]^{2+}$ *m/z* 475.20 (Figure S15); for iRGD peptide observed $[M + H^+]^+$ *m/z* 947.38 and $[M + 2H^+]^{2+}$ *m/z* 474.19 (Figure S16).

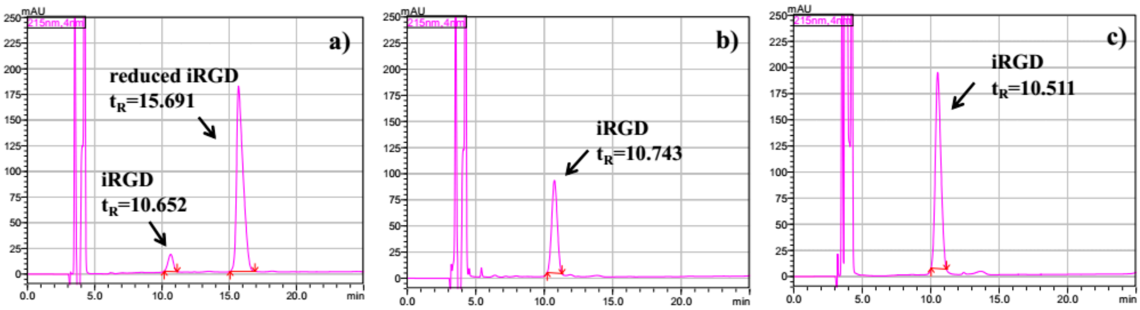

Figure S14. HPLC chromatograms of (**a**) reduced iRGD (a disulfide-based cyclic RGD peptide, c(CRGDKGPDC)) after stirring for 30 min; (**b**) supernatant from the reaction of SiO_2@TPEA@Pt(IV) with reduced iRGD for 30 min; and (**c**) $[Pt(en)_2Cl_2]Cl_2$ and reduced iRGD reaction mixture.

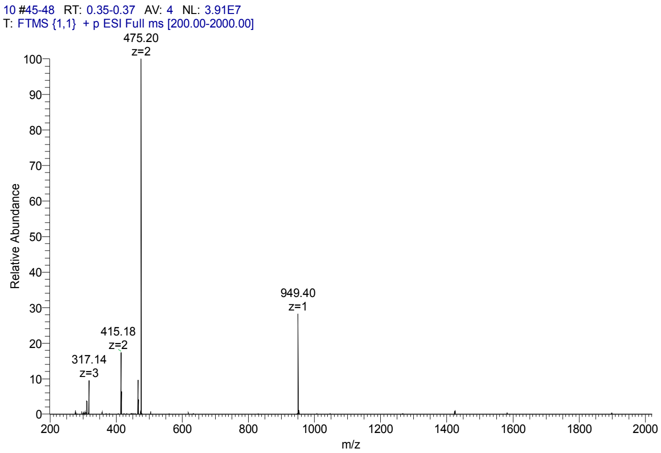

Figure S15. MS of reduced iRGD.

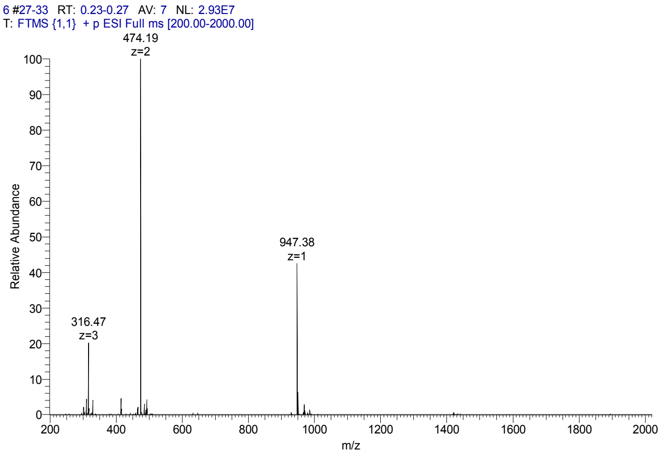

Figure S16. MS of iRGD.

4.5. HPLC and ESI-MS characterization of reduced oxytocin

Reduced oxytocin was dissolved in a pH 4.5 buffer (2 mg/mL, 1.2 mL) and stirred at 25 °C for 30 min. The solution was then analyzed by a gradient HPLC equipped with a UV-vis detector at 215 nm using a 250 mm × 4.6 mm C_8 column at a flow rate of 1.0 mL/min at room temperature. The HPLC solvent was composed of solvent A (0.03% TFA in acetonitrile) and solvent B (0.03% TFA in water). The elution protocol for analytical HPLC started with 90% B, followed by a linear gradient to 25% B over 10 min, and further to 50% B over 10 min, held at 50% B for 5 min, continued to 90% B over 20 min, and finally returned to 90% over 10 min. The HPLC chromatogram obtained is shown in Figure S17a.

Next, reduced oxytocin was dissolved in a pH 4.5 buffer (2 mg/mL, 1.2 mL) and reacted with SiO_2@TPEA@Pt(II) (40 mg) or SiO_2@TPEA@Pt(IV) (40 mg) under stirring at 25 °C for 30 min. After centrifugation of the mixture, the supernatants obtained were analyzed using the same HPLC method as described above. The HPLC chromatogram obtained is shown in Figure S17b and S17c.

Reduced oxytocin was then dissolved in pH 4.5 buffer (2 mg/mL, 1.2 mL) and reacted with $[Pt(en)_2Cl_2]Cl_2$ (1.5 mg) at 25 °C for 30 min. The mixture was analyzed using the HPLC method described above, and the result is shown in Figure S17d.

Reduced oxytocin and oxytocin were characterized by ESI-MS in the positive mode; for reduced oxytocin observed $[M + H^+]^+$ *m/z* 1009.46 and $[M + Na^+]^+$ *m/z* 1031.44 (Figure S18); for oxytocin observed $[M + H^+]^+$ *m/z* 1007.44 and $[M + Na^+]^+$

m/z 1029.42 (Figure S19).

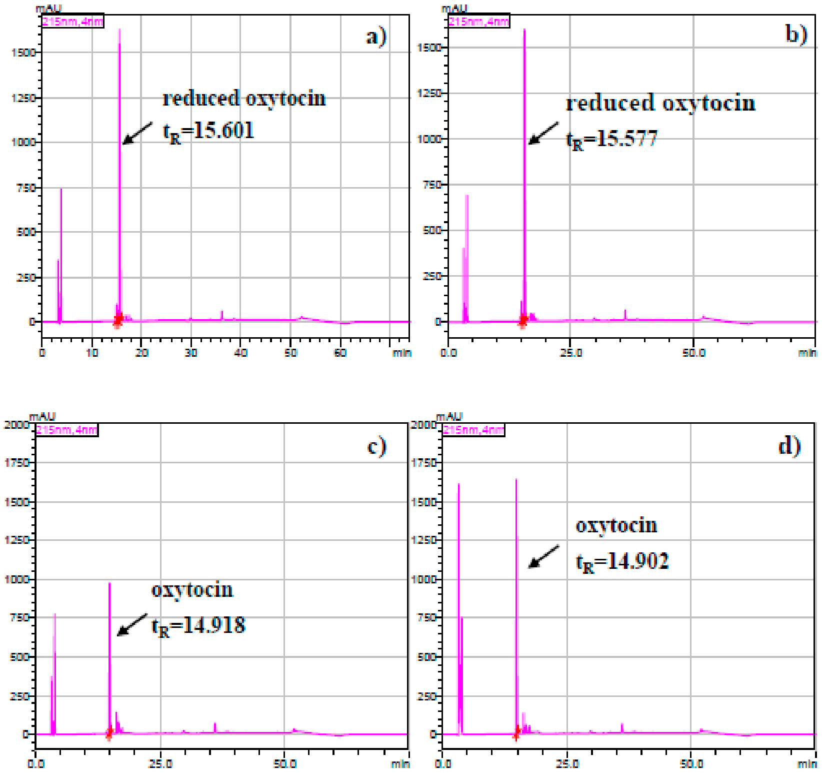

Figure S17. HPLC chromatograms of (**a**) reduced oxytocin after stirring for 30 min; (**b**) supernatant from the mixture of SiO_2@TPEA@Pt(II) with reduced oxytocin stirred for 30 min; (**c**) supernatant from the reaction mixture of SiO_2@TPEA@Pt(IV) with reduced oxytocin for 30 min; and (**d**) mixture of $[Pt(en)_2Cl_2]Cl_2$ with reduced oxytocin.

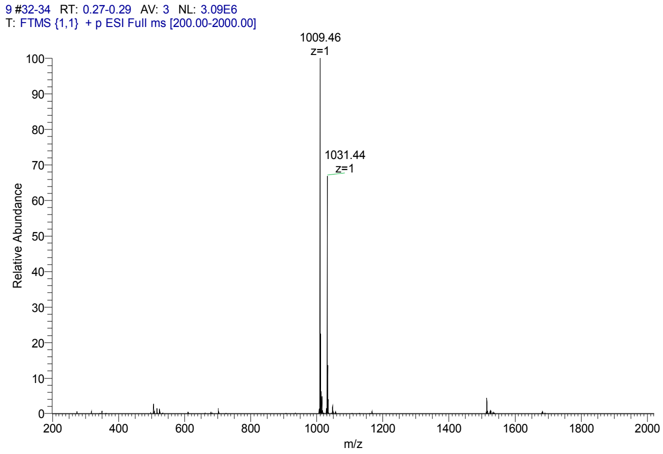

Figure S18. MS of reduced oxytocin.

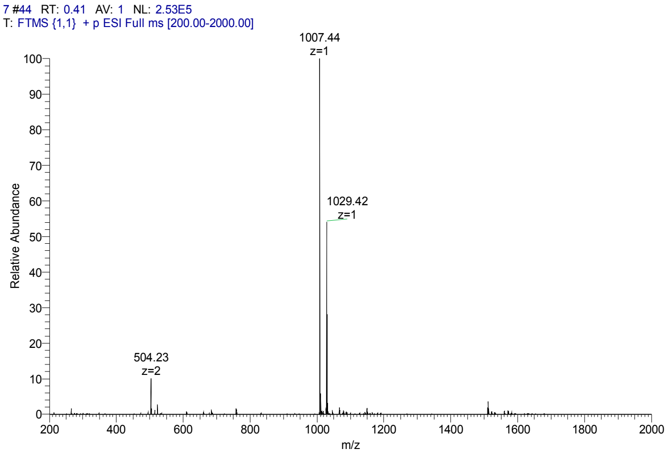

Figure S19. MS of oxytocin.

4.6. HPLC and ESI-MS characterization of reduced somatostatin

Reduced somatostatin was dissolved in a solution containing pH 4.5 buffer and acetonitrile (3:1 *v/v*) (1 mg/mL, 2 mL) and stirred at 25 °C for 30 min. The solution was then analyzed by a gradient HPLC equipped with a UV-vis detector at 215 nm using a 250 mm × 4.6 mm C_8 column at a flow rate of 1.0 mL/min at room temperature. The HPLC solvent mixture consisted of solvent A (0.1% TFA in acetonitrile) and solvent B (0.1% TFA in water). The elution protocol for the HPLC analysis started with 70% B, followed by a linear gradient to 0% B over 15 min, and returned to 70% over 5 min. The HPLC chromatogram obtained is shown in Figure S20a.

Next, reduced somatostatin solution (1 mg/mL, 2 mL) was reacted with SiO_2@TPEA@Pt(IV) (25 mg) or $[Pt(en)_2Cl_2]Cl_2$ (1.0 mg) under stirring at 25 °C for 30 min. In the former case, the mixture was subjected to centrifugation and the supernatant obtained was analyzed using HPLC. In the latter case, the reaction mixture was analyzed using HPLC. The conditions described above were used for the HPLC analyses, and the results for the two cases are shown in Figure S20b and Figure S20c, respectively.

Reduced somatostatin and somatostatin were characterized by ESI-MS in the positive mode; for reduced somatostatin observed $[M + 2H^+]^{2+}$ *m/z* 820.37 and $[M + H^+]^+$ *m/z* 1639.74 (Figure S21); for somatostatin observed $[M + 2H^+]^{2+}$ *m/z* 819.37 and $[M + 3H^+]^{3+}$ *m/z* 546.58 (Figure S22).

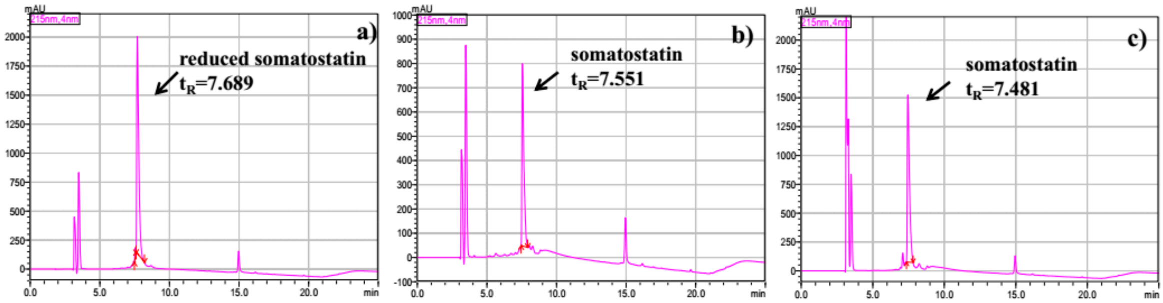

Figure S20. HPLC chromatograms of (**a**) reduced somatostatin after stirring for 30 min; (**b**) supernatant from the reaction of SiO_2@TPEA@Pt(IV) with reduced somatostatin for 30 min; and (**c**) $[Pt(en)_2Cl_2]Cl_2$ and reduced somatostatin reaction mixture.

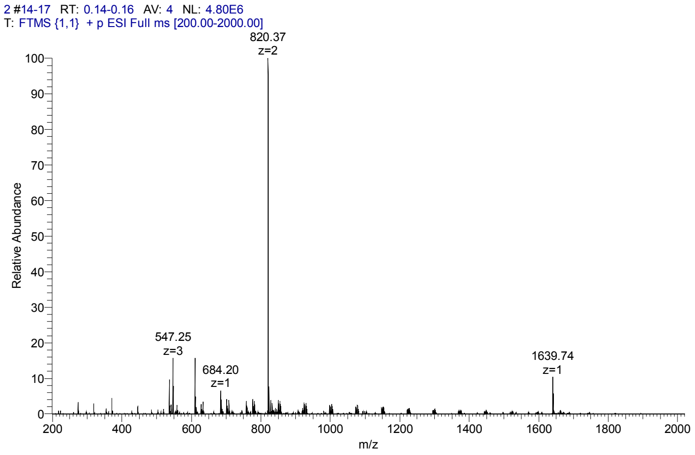

Figure S21. MS of reduced somatostatin.

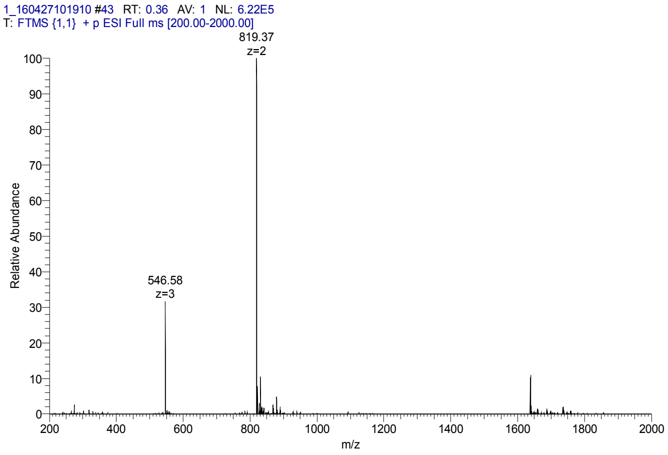

Figure S22. MS of somatostatin.

4.7. HPLC analysis of the interaction of oxytocin with SiO_2@TPEA@Pt(II)

Reduced oxytocin was dissolved in pH 4.5 buffer (2 mg/mL, 3.3 mL) and reacted with 4.4 mg of $[Pt(en)_2Cl_2]Cl_2$ for 30 min. The mixture was then analyzed by HPLC using the protocol described in section 3.5. The HPLC chromatogram is shown in Figure S23a. Further, the above solution (1.2 mL) was mixed with 40 mg of SiO_2@TPEA@Pt(II) under stirring for 30 min. The mixture was centrifuged, and the supernatant was analyzed by HPLC. The result is shown in Figure S23b.

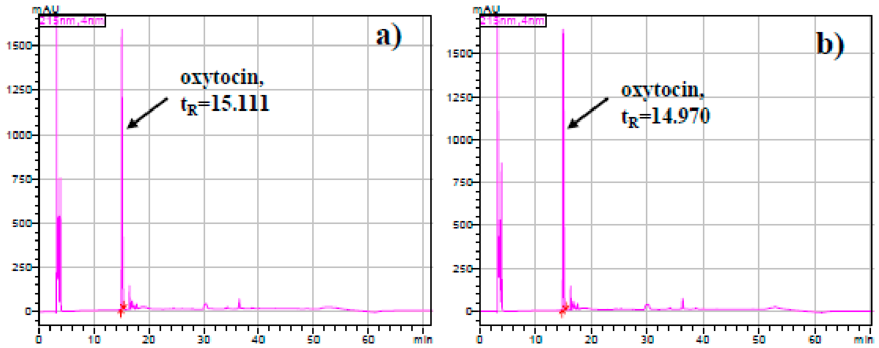

Figure S23. HPLC chromatograms of (**a**) the reaction mixture of $[Pt(en)_2Cl_2]Cl_2$ with reduced oxytocin for 30 min; and (**b**) supernatant from the reaction mixture of SiO_2@TPEA@Pt(II) with 1.2 mL of (a) for 30 min.
